# Severe tracheal stenosis due to prolonged tracheostomy tube placement: a case report

**DOI:** 10.4076/1757-1626-2-7101

**Published:** 2009-09-18

**Authors:** Wei Zhou, Shi Fang Ding, Qian Zhai, Da Wei Wu

**Affiliations:** 1 Department of Radiology, Shandong University Qilu HospitalJinan, Shandong, 250012China; 2 Department of Intensive Care unit, Shandong University Qilu HospitalJinan, Shandong, 250012China

## Abstract

Tracheal stenosis is the most common late airway complication of tracheostomy. Severe tracheal stenosis resulted in hemodynamic deterioration and impairment of respiratory system mechanics. We cared for an 86-year-old man with severe tracheal stenosis due to prolonged placement of a tracheostomy tube for 42-months. At the distal tip of the tracheostomy tube, bronchoscopy revealed severe tracheal luminal obstructions by granulation tissue. During pressure-controlled ventilation, the peak airway pressure was much higher than the inspiratory pressure. For patients with clinical signs of tracheal stenosis after tracheotomy, bronchoscopy should be done as early as possible.

## Introduction

Severe tracheal stenosis can be life-threatening when critical narrowing of the airway occurs [1,2]. However, identification of tracheal stenosis may be particularly challenging due to the insidious development and the patient’s co-existent severe illness. Fiberoptic bronchoscopy is the gold standard for identification of tracheal stenosis [3]. We recently cared for a patient with severe tracheal stenosis after a tracheostomy *in situ* for nearly 4 years.

## Case presentation

A 86-year-old Han Chinese man had a hypertensive intraventricular hemorrhage on 7 December 2004. He immediately underwent emergency surgical placement of bilateral external ventricular drains under general anesthesia, and was transferred to the intensive care unit (ICU) for mechanical ventilation. After 3 days, a standard surgical tracheostomy was performed because the patient had a short thick neck. A high-volume, low-pressure cuffed tracheostomy tube with an 8.0 mm ID and an 11.0 mm OD was inserted. On 20 December 2004, he was weaned off of ventilatory support. The patient remained deeply comatose and descended into a persistent vegetative state. His stay in the ICU with aggressive nursing measures continued.

On 25 June 2007, the patient had progressive respiratory distress with rapid shallow breathing, while using all accessory muscles of respiration. He had a respiratory rate of 50 breaths.min^-1^, and a fluctuating arterial oxygen saturation of 70~80% with 10 L.min^-1^ of supplementary oxygen. The patient was ventilated with the synchronized intermittent mandatory ventilation (SIMV) pressure-controlled mode. The settings were as follows: respiratory rate (RR), 16 breaths/min; inspiratory pressure, 16 cm H_2_O; inspiratory time, 0.9 second; pressure support (PS), 16 cm H_2_O; positive end expiratory pressure (PEEP), 3 cm H_2_O; and FiO_2_, 0.35. With these settings, the peak airway pressure (PIP) was 19 cm H_2_O, the exhaled tidal volume was 600 ml, and the arterial blood gas parameters were satisfactory. He was weaned off of mechanical ventilatory support after 9 days. On 14 January 2008, he experienced a similar episode of dyspnea and received intermittent mechanical ventilation for 5-15 d at 1-2 month intervals. The patient’s dyspnea worsened progressively, requiring ongoing ventilator dependency, accompanied with PIP, which was gradually increased to 35~45 cm H_2_O.

**Figure 1.**
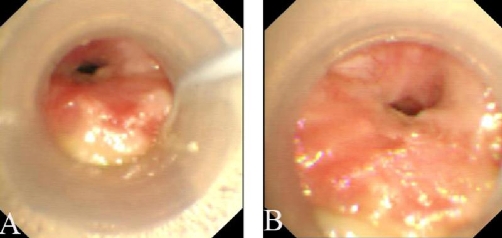
Typical bronchoscopic photographs showed the tracheal lumen almost completely obstructed during inspiration (panel A) and expiration (panel B) by granulation tissue arising from the posterior wall.

On 24 May 2008, while endotracheal secretions were being suctioned, it became difficult to insert a single-use silicone catheter through the endotracheal tube. The catheter could not be passed, despite instillation of saline to lubricate the catheter and various twisting catheter maneuvers. When disconnecting from the ventilator, the patient’s spontaneously breathe showed very low gas flow both inspiration and expiration (Movie 1). During pressure-controlled ventilation, the PIP sometimes reached 60~80 cm H_2_O, accompanied with biphasic stridor heard (Movie 2), an exhaled tidal volume ≤ 200 ml, and CO_2_ retention (arterial blood gas analysis: pH 7.21, PaCO_2_ 82.0 mm Hg, PaO_2_ 98 mm Hg). The patient was suspected of having tracheal stenosis. On 4 June 2008, bronchoscopy was performed emergently through the tracheostomy tube. At the distal tip of the tracheostomy tube, the bronchoscopy revealed severe tracheal luminal obstruction by granulation tissue (Figure 1). The bronchoscope could not be passed through the stenosed site (5.2 mm OD), therefore the ventilatory settings were changed to volume-controlled mode as follows: tidal volume, 480 ml; RR, 16 breaths.min^-1^; peak inspiratory rate, 40 L.min^-1^; PS, 16 cm H_2_O; and FiO_2_, 0.35. This mode improved the arterial blood gases values gradually with the PIP ranging between 40 and 55 cm H_2_O. On 16 June 2008, the patient’s daily urine output decreased to < 400 ml (sometimes < 100 ml), while serum creatinine and BUN levels progressively rose to 469 μmol/l and 41.6 mmol/l, respectively. Continuous renal replacement therapy was performed every other day. Therapeutic approaches, such as tracheal sleeve resection and endotracheal tube insertion, were not performed since the patient’s authorized guardian declined. The patient died on 18 August 2008.

## Discussion

Tracheal stenosis is the most common late airway complication of tracheostomy [1]. In the patient presented herein, tracheal stenosis occurred at the site of the tracheostomy tube’s distal tip by granulation tissue [4]. There are multiple risk factors associated with tracheal stenosis. Prolonged placement of a tracheostomy tube accounts for 90% of cases [1]; in the patient described herein, the tracheostomy tube was in place for 42 months. Second, the patient coughed up airway secretions and underwent suctioning frequently, which resulted in excessive tube motion and irritation. Third, the patient’s short, thick neck made the tracheostomy tube too short, and the tube tip rubbed against the tracheal membranous wall, resulting in injury, granulation tissue formation, and an obstructed tracheal lumen.

Severe tracheal stenosis can also create intrinsic positive end expiratory pressure, increase the patient’s effort to breathe, especially in patients with asthma or chronic obstructive pulmonary disease, and limit weaning from the respirator [5]. For this patient, he had to rely on mechanical ventilation to prolong his life. Moreover, severe tracheal stenosis can deteriorate cardiac output through increased airway pressure and intrathoracic pressure [6]. Therefore, the reduced cardiac output affects systemic and renal hemodynamics, which is one mechanism for mechanical ventilation initiation or aggravation acute renal failure [6,7]. We think that the higher PIP contributed to the patient’s renal failure.

As is known, the PIP should be equal to the level of inspiratory pressure in the pressure-controlled ventilation mode. In this patient, however, the PIP progressively increased to 35~45 cm of H_2_O, even though the inspiratory pressure was 16 cm H_2_O during the pressure-controlled ventilation mode. As Brouns et al. [7] reported, the pressure drop over tracheal stenosis dramatically increased dependence on the severity of stenosis. During pressure-controlled ventilation, the peak airway pressure was much higher than the inspiratory pressure because of the pressure drop over severe tracheal airway stenosis; this may explain a rare abnormal phenomenon. The ventilatory strategies for severe tracheal stenosis should include volume-controlled ventilation, tidal volume, respiratory rate, and an inspiratory peak flow at low values, according to the pressure drop over tracheal stenosis depending on inspiratory flow [7,8].

However, the diagnosis of tracheal stenosis is often made until the tracheal lumen has been reduced by 75%-90% and subsequent endanger the patients’ life at any time. There are three explanations for the delayed diagnosis of this patient’s tracheal stenosis: 1) the rarity of severe stenosis and its atypical respiratory symptoms, 2) the patients have other co-existing severe illnesses and the physicians lack suspicion of the possibility of tracheal stenosis, and 3) bronchoscopy, which is the most reliable method for identification of tracheal stenosis, is not routinely performed in patients with a history of intubation or tracheostomy in China [3]. For patients with clinical signs of tracheal stenosis, a number of protocolized approaches have been recommended, and the treatment often required as a semi-emergency [1,10].

In conclusion, severe tracheal stenosis resulted in higher PIP and hemodynamic deterioration. For patients with clinical signs of tracheal stenosis after tracheotomy, bronchoscopy should be done as early as possible.

## Additional files

Movie 1: When disconnecting from the ventilator, the patient’s spontaneously breath showed very low gas flow with stridor both inspiration and expiration. http://casesjournal.com/casesjournal/article/downloadSuppFile/7101/21148

Movie 2: Biphasic stridor heard during mechanical ventilation. http://casesjournal.com/casesjournal/article/downloadSuppFile/7101/21149

## Abbreviations

ICU: intensive care unit
PEEP: positive end expiratory pressure
PIP: peak airway pressure
PS: pressure support
RR: respiratory rate
SIMV: synchronized intermittent mandatory ventilation
